# A Review of Anti-Inflammatory Drug-Induced Gastrointestinal Injury: Focus on Prevention of Small Intestinal Injury

**DOI:** 10.3390/ph3041187

**Published:** 2010-04-20

**Authors:** Shunji Fujimori, Katya Gudis, Choitsu Sakamoto

**Affiliations:** Department of Internal Medicine, Division of Gastroenterology, Nippon Medical School 1-1-5, Sendagi, Bunkyo-ku, Tokyo 113-8603, Japan

**Keywords:** NSAID, aspirin, small intestine, injury, misoprostol, rebamipide

## Abstract

Capsule endoscopy and balloon endoscopy, advanced modalities that allow full investigation of the entire small intestine, have revealed that nonsteroidal anti-inflammatory drugs (NSAIDs) can cause a variety of abnormalities in the small intestine. Recently, several reports show that traditional NSAIDs (tNSAIDs) and acetylsalicylic acid (ASA) can induce small intestinal injuries. These reports have shown that the preventive effect of proton pump inhibitors (PPIs) does not extend to the small intestine, suggesting that concomitant therapy may be required to prevent small intestinal side effects associated with tNSAID/ASA use. Recently, several randomized controlled trials used capsule endoscopy to evaluate the preventive effect of mucoprotective drugs against tNSAID/ASA-induced small intestinal injury. These studies show that misoprostol and rebamipide reduce the number and types of tNSAID-induced small intestinal mucosal injuries. However, those studies were limited to a small number of subjects and tested short-term tNSAID/ ASA treatment. Therefore, further extensive studies are clearly required to ascertain the beneficial effect of these drugs.

## Introduction

**Figure 1:**
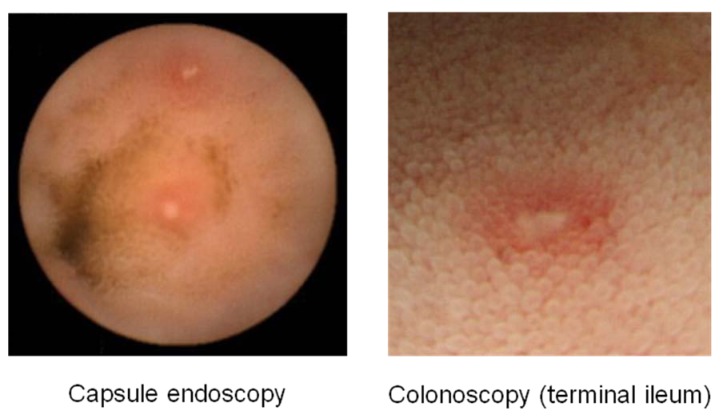
Traditional NSAIDs-induced small intestinal mucosal breaks (erosions).

**Figure 2:**
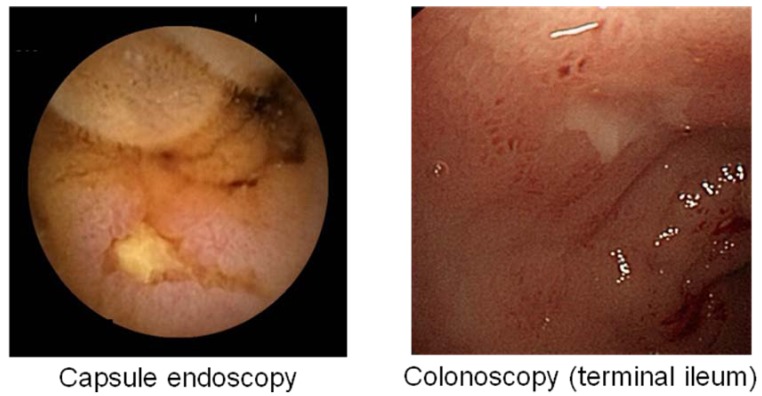
ASA-Induced small intestinal mucosal breaks (ulcers).

**Figure 3:**
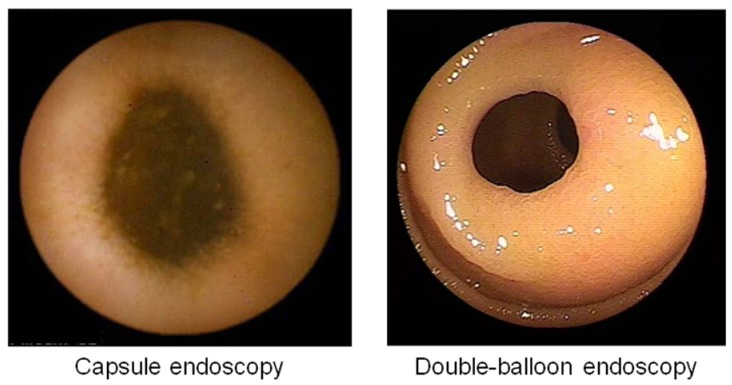
Traditional NSAIDs-induced diaphragm-like stricture.

Nonsteroidal anti-inflammatory drugs (NSAIDs) are among the most commonly prescribed drugs worldwide. They are widely used to help relieve musculoskeletal pain and inflammation, but can cause serious upper gastrointestinal side effects including dyspepsia, peptic ulceration, and hemorrhage. These adverse events have been shown to occur in approximately 1-1.5% of patients within the first 12 months of treatment with traditional NSAIDs (tNSAIDs) [1,2]. tNSAID-induced gastrointestinal adverse events have been shown to cause death in some cases [3]. It has been estimated that in 1998 there were 16,500 cases of NSAID-included fatalities in the U.S. alone due to acetylsalicylic acid (ASA) toxicity [4]. Another large study in Spain reported 15.3 deaths out of 100,000 NSAID users including aspirin [5]. Until recently, most studies on tNSAID/ASA-associated injury have focused on the upper gastrointestinal tract, since the stomach and duodenum are the sites generally associated with major morbidity and mortality in the clinical setting. Therefore, proton pump inhibitors and prostaglandin analog have become the established treatment against tNSAID/ASA-induced gastroduodenal injuries [6,7]. However, epidemiological studies suggest that NSAIDs may also increase the risk of lower gastrointestinal adverse events [8,9]. One recent prospective trial showed that serious lower gastrointestinal events in rheumatoid arthritis patients taking NSAIDs may account for 40% of all serious gastrointestinal events that develop in these patients [10]. In addition, capsule endoscopy and double-balloon endoscopy [11,12], advanced modalities that now allow for full investigation of the entire small intestine, have revealed that tNSAID/ASA can cause a variety of abnormalities in the small intestine; such as erosions, ulcerations, perforation, bleeding and diaphragm-like stricture (Figure 1, Figure 2 and Figure 3) [8,9,13,14,15,16,17,18].

Lanas *et al*. report that the incidence of upper gastrointestinal complications is decreasing while the incidence of lower gastrointestinal complications is increasing; the rate of upper/lower gastrointestinal complications was 4.1 in 1996 and 1.4 in 2005 [19]. Recently, clinical studies have been trying to determine whether prostaglandin analog and mucoprotective drugs are as effective against tNSAID/ASA-induced small intestinal injury as has been shown for gastroduodenal injuries. Several drugs have been evaluated for their ability to reduce tNSAID/ASA-induced small intestinal injuries.

## Prevalence of tNSAID/ASA-Induced Small Intestinal Injury

### Traditional NSAIDs

Indium-111-labeled white blood scintigraphy detected small intestinal inflammation in more than 50% of chronic tNSAID users, and fecal tests found signs of intestinal permeability and inflammation in 44% of this class of patients [20]. Morris *et al.* showed ulcerations by sonde enteroscopy in 7 of 15 (47%) rheumatoid arthritis patients on tNSAID medication [16]. In Japan, small intestinal mucosal breaks were detected by double-balloon endoscopy in 51% of NSAIDs users *versus* 5% in those not taking NSAIDs [15]. In a recent study, Maiden *et al.* found new intestinal lesions by capsule endoscopy in 68% of healthy volunteers who took tNSAIDs for two weeks [21]. Goldstein *et al.* reported that 55% of subjects developed small intestinal injuries after two weeks of naproxen medication, with a mean of 2.99 mucosal breaks per subject [22]. Japanese studies support these findings, showing that over 50% of subjects developed small intestinal mucosal breaks after two weeks of diclofenac medication [23,24,25]. These studies suggest that tNSAIDs cause small intestinal injuries in over 50% of subjects. 

### Acetylsalicylic acid (ASA)

Slattery *et al.* recruited 2,435 patients for their UK-TIA trial to analyze the effect of ASA on lower gastrointestinal bleeding, defined as fresh blood per rectum, and reported odds ratios of 1.8 (0.5 to 6.1) and 1.5 (0.4 to 5.3) for ASA doses of 300 mg and 1,200 mg, respectively [26]. A thrombosis prevention trial published in the journal Lancet in 1998 reported a higher incidence of rectal bleeding in ASA users (10.0%; 127/1268: 8105 person years) than in non ASA users (7.5%; 96/1272: 8071 person years) [27]. Recently, a number of studies have been published that used capsule endoscopy to evaluate for ASA-induced small intestinal injuries. Watanabe *et al*. detected small intestinal injuries in all eleven patients who underwent low-dose enteric-coated ASA therapy, using capsule endoscopy [18]. Endo *et al*. also reported that small intestinal pathologies were more prevalent in patients on two weeks of low-dose ASA medication than in patients taking placebo. In that study, minor lesions developed in 80% of subjects on ASA medication *versus* 20% in the control group, and small intestinal mucosal breaks developed in 30% of ASA users *versus* 0% in the non-users [28]. Shiotani *et al*. reported that small intestinal mucosal breaks were detected in 90% of healthy subjects after only a week of low-dose enteric-coated ASA medication, a much higher rate than reported by others [29]. In sum, there has been such a wide variation among reports that it is difficult to form a clear picture of the prevalence of ASA-induced small intestinal mucosal injuries. Further extensive studies are clearly required to determine the effect of ASA on small intestinal mucosal injuries.

## Key Process of tNSAID/ASA-Induced Small Intestinal Injury

NSAIDs are known to increase intestinal permeability, the magnitude of which directly correlates to the potency of their ability to inhibit cyclooxygenase-1 (COX-1) [30,31]. Inhibition of COX-1 reduces levels of protective mucosal prostaglandins in the small intestine [32]. The precise mechanism by which the inhibition of COX by NSAIDs translates into injury of the small intestine is poorly understood. Nevertheless, the first step leading to small intestinal mucosal injury is considered to be the topical toxicity of NSAIDs, which induces the uncoupling of mitochondrial oxidative phosphorylation in epithelial cells [33]. This topical action is followed by increased mucosal permeability and inflammation [34], which appears to be a prerequisite for NSAID-induced small intestinal injury and ulceration. However, it has been clearly shown that COX-1 inhibition is also required to convert topical toxicity into ulcerative damage. 

Somasundaram *et al.* have shown that co-administration of ASA, a COX-1 inhibitor that is mainly absorbed through the stomach and duodenum; and dinitrophenol, which increases intestinal permeability through the disruption of mitochondrial activity, induces intestinal ulceration similar to that induced by indomethacin [34]. Meanwhile, transgenic COX-1 knockout mice have no apparent intestinal pathology and are less sensitive to tNSAID-induced ulceration [35]. Small intestinal damage (NSAID enteropathy) is set off by a synergistic action of two or more of the biochemical actions common to all tNSAIDs (COX-1+COX-2 inhibition, COX-1 inhibition +“topical” effect, *etc.*) [36]. Topical effects include effects by luminal contents such as luminal bacteria, bile, food and enzyme, changes of intestinal motility, *etc.* [31,36,37]. Thus small intestinal injury is not induced by only COX-1 inhibition. But, previous data suggest that the inhibition of COX-1 is likely to be a key process in intestinal ulceration.

## COX-2 Inhibitors or Proton Pump Inhibitors against Small Intestinal Injury

Capsule endoscopy studies have shown that even concomitant administration of PPIs failed to prevent tNSAID-induced small intestinal injury in healthy volunteers [21, 22]. As for the prevention of NSAID-induced small intestinal injury, several studies have already shown that celecoxib, a selective COX-2 inhibitor, effectively reduces both the number of mucosal breaks per subject and the percentage of subjects with at least one mucosal break [22, 38]. COX-2 inhibitors were initially introduced to provide symptomatic pain relief along with reduced gastrointestinal risk. 

However, in 2005, a joint hearing of the US Food and Drug Administration Arthritis Committee, and the Drug Safety and Risk Management Committee found that the use of COX-2 inhibitors is associated with increased risk of cardiovascular events. The current thought is that the cardiovascular risk of COX-2 inhibitors is the same as that of tNSAIDs. This has led many physicians to consider the use of tNSAIDs in combination with a proton pump inhibitor, a recommendation found in major treatment guidelines for patients with a history of gastrointestinal events or for those at high risk of developing complications [9]. Indeed, many physicians are again using tNSAIDs in combination with proton pump inhibitors as the preferred preventive method against tNSAID-induced gastrointestinal injury [39, 40]. However, studies have shown that the preventive effect of proton pump inhibitors does not extend to the small intestine, suggesting that concomitant therapy may be required to prevent small intestinal side effects associated with tNSAID use. The most recent studies on the prevention of tNSAID-induced small intestinal injuries are discussed in the section that follows. 

## Studies Evaluating the Preventive Effect of Certain Drugs against NSAID/ASA Toxicity in Small Intestine

### Prostaglandin analog (misoprostol)

It has been suggested that NSAID-induced inhibition of COX-1, a key molecule that catalyzes prostaglandin (PG) production, is involved in the disruption of the protective mechanism in the gastric mucosa [39]. It is widely known that PG is effective in preventing NSAID-induced gastric mucosal injury [40,41,42]. As for NSAID-induced small intestinal injuries, a sequence of events, such as an increase in the permeability of epithelial cells due to the direct toxic effect of NSAIDs, bacterial translocation, and inflammation through cytokine activation in the small intestinal mucosa, have been suggested to be key elements in the induction of small intestinal ulceration in addition to a lack of prostaglandin [33,34,43]. As for injury to the small intestine, PG has been shown to reverse NSAID-induced changes in intestinal permeability, a local intestinal event that is considered to play a pivotal role in inflammation and injury [44]. Furthermore, the co-administration of misoprostol, a PGE1 analog, has been shown to attenuate the effect of NSAIDs on intestinal permeability in humans [44]. Therefore, our own group investigated the effect of misoprostol on small intestinal injury induced by tNSAID (diclofenac) in a single-blind, randomized controlled study [23].

**Figure 4:**
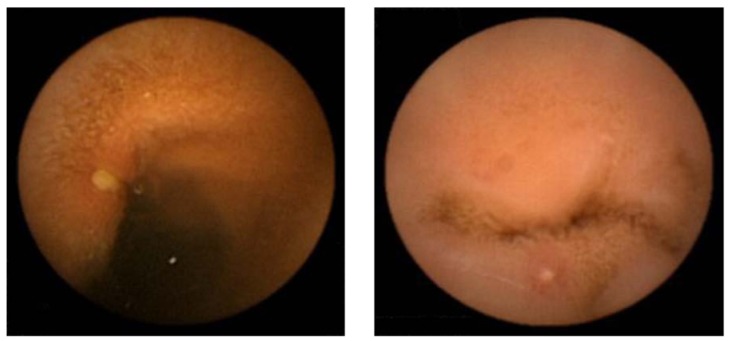
Example of typical mucosal breaks found in the study.

In that study, thirty-four healthy male volunteers were screened by capsule endoscopy. All eligible subjects (n = 32) were randomly divided into a control group (n = 16) and a PG group (n = 16). All eligible subjects were administered diclofenac (75 mg/day) and omeprazole (20 mg/day) for a period of two weeks, and the PG group assigned to receive misoprostol (600 μg/day) in addition to the original treatment. We defined mucosal breaks in the small intestine as lesions with slough surrounding erythema and calculated their incidence. Examples of typical mucosal breaks are shown in Figure 4.

A total of 15 control subjects and 15 PG subjects completed the treatment; the entire small intestine of each subject was evaluated by capsule endoscopy. In the control group, two weeks of treatment induced 44 mucosal breaks in eight subjects, resulting in a mean of 2.9 ± 6.3 mucosal breaks per subject. In the PG group, PG treatment reduced the number of mucosal breaks to 10 in two subjects (mean = 0.7 ± 2.3). Thus, at post-treatment capsule endoscopy, the mean number of mucosal breaks per subject was significantly higher in the control group than in the PG group (p = 0.028). The percentage of subjects with at least one mucosal break at post-treatment was also significantly higher in the control group (53.3%) than in the PG group (13.3%) (p = 0.002). Three subjects in the PG group and one subject in the control group complained of slight diarrhea at the beginning of treatment [23]. 

In our study, misoprostol was effective in preventing the development of small intestinal mucosal breaks in healthy individuals receiving a two-week regimen of diclofenac. The percentage of subjects that were found to have mucosal breaks at baseline endoscopy was 10%, compared to reports of 7-14% in other studies, indicating that co-administration of misoprostol can reduce the development of mucosal breaks in patients on diclofenac medication down to approximately basal levels [22,38,45]. Taking into consideration previous reports and our data, it appears that misoprostol can prevent both upper and small intestinal injuries associated with the use of tNSAIDs.

Watanabe *et al.* reported that misoprostol prevented low-dose enteric-coated ASA-induced small intestinal injuries in four out of seven patients [18]. Their study enrolled 11 patients who developed gastric ulcers while undergoing low-dose enteric-coated ASA therapy. Patients continued ASA therapy while taking proton pump inhibitors for eight weeks to heal the gastric ulcers. Then misoprostol 200 μg four times a day was administered for eight weeks instead of proton pump inhibitors. Capsule endoscopy performed after eight weeks of proton pump inhibitor treatment identified red spots and mucosal breaks in 100% (11/11) and 90.9% (10/11) of patients, respectively. In seven patients who completed the study protocol, misoprostol significantly decreased the mean number of red spots and mucosal breaks, with complete disappearance of mucosal breaks in four patients. Intestinal lesions tended not to heal in three patients who discontinued misoprostol due to diarrhea, a side effect of misoprostol. The single remaining patient was dropped out of the study because the patient discontinued ASA treatment. 

Together with our study, these results suggest that misoprostol can prevent not only tNSAID-induced small intestinal injuries but also those induced by the ingestion of ASA. 

### Rebamipide

As previously mentioned, the authors have shown that co-administration of misoprostol reduced the incidence of small intestinal lesions induced by two-week administration of diclofenac [23]. However, misoprostol can induce intolerable side effects as reported previously [46]. Rebamipide has been used across Asia for the treatment of gastric ulcers and gastric lesions such as erosions and edema caused by acute gastritis [46,47,48]. It has been well documented that rebamipide increases endogenous prostaglandin levels, scavenges free radicals, and suppresses inflammation in the gastric mucosa [20, 49, 50]. Through these actions, rebamipide has been also shown to be useful in preventing NSAID-induced gastrointestinal injuries in clinical studies and animal experiments. In a randomized controlled trial of rheumatoid arthritis and osteoarthritis patients carried out in East Asian countries, the effectiveness of rebamipide was shown to equal that of misoprostol in preventing the incidence ratio of gastroduodenal ulcers caused by 12 weeks of tNSAIDs medication [49]. In an animal experiment, rebamipide was shown to inhibit increases in iNOS activity induced by indomethacin, thereby reducing small intestinal injury caused by tNSAIDs in rats [51]. From all these data, it is reasonable to speculate that to some extent, rebamipide might serve to reduce small intestinal damage in patients on NSAID medication. 

A preliminary study recently conducted by Niwa *et al.* has shown that rebamipide effectively reduced the incidence of diclofenac-induced small intestinal injury [25]. Their positive data was obtained in a double-blind, randomized, cross-over study where subjects were treated with diclofenac (75 mg/day) and omeprazole (20 mg) in the presence or absence of rebamipide (300 mg/day) for seven days. The study shows that the number of subjects with small intestinal mucosal injuries was higher in the placebo group (8/10) than in the rebamipide group (2/10) (P = 0.023). Unfortunately, the study was underpowered with the analysis of only 10 subjects. Therefore, we conducted our own study using a larger number of patients to re-evaluate the effect of rebamipide on diclofenac-induced small intestinal injuries in healthy subjects, in a double-blind, randomized controlled trial [24]. 

**Figure 5:**
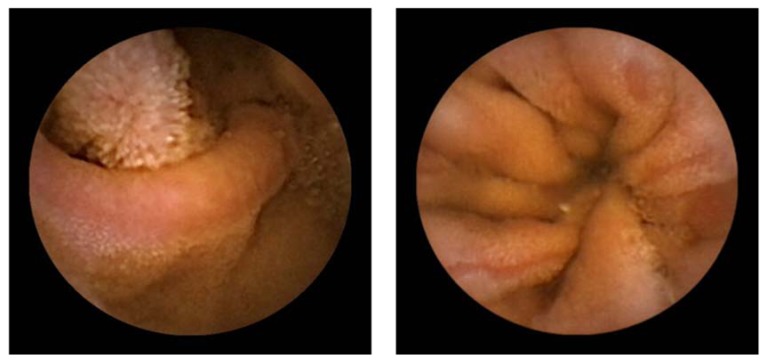
Example of typical denuded areas found in the study.

Eighty healthy male volunteers were randomly divided into a placebo group (n = 40) and a rebamipide group (n = 40). After evaluation by baseline capsule endoscopy, all eligible subjects were administered diclofenac and omeprazole for a period of two weeks. The placebo group was assigned to remain on the original diclofenac and omeprazole therapy with a placebo capsule, while the rebamipide group was assigned to receive a capsule filled with rebamipide in addition to the original treatment. These doses were the same tested in a previous study. A total of 38 control subjects and 34 rebamipide subjects completed the treatment and were evaluated by capsule endoscopy. Diclofenac therapy increased the mean number of mucosal injuries per subject, from a basal level of 0.1 ± 0.3, to 15.9 ± 71.6 and 4.2 ± 7.8 in the control and rebamipide co-treatment groups, respectively. The difference between those two groups was not significant. Mucosal injuries consisted of both mucosal breaks (Figure 4) and denuded areas (Figure 5) in this study. These two types of lesions are not associated with one another [52]. 

The difference in the percentage of subjects with at least one mucosal injury at post-treatment was also not significant (control, 63%; rebamipide, 47%). However, when we limited our analysis to subjects with mucosal injuries, rebamipide co-treatment significantly reduced the mean number of mucosal injuries per subject, from 25.1 ± 89.3 in the placebo group to 8.9 ± 9.4 in the rebamipide group (p = 0.038). We found that rebamipide reduced the intensity of injury in subjects apparently susceptible to diclofenac-induced small intestinal injuries. These data were presented at the Digestive Disease Week 2009 conference in Chicago [24].

### Other drugs

Metronidazole and sulfasalazine were evaluated for their preventive effect against tNSAID-induced small intestinal inflammation [53,54]. Bjarnason *et al.* reported that metronidazole 800 mg/day reduced intestinal permeability, blood loss, and inflammation in 32 patients with continuous tNSAID treatment. Intestinal inflammation, as assessed by the faecal excretion of indium-111 labeled neutrophils, and blood loss, assessed with chromium-51 labelled red cells, were both significantly reduced after metronidazole treatment [55]. This same group reported that sulfasalazine also reduced both intestinal inflammation and blood loss in 46 patients receiving tNSAIDs [56].

Niwa *et al.* also reported that geranylgeranylacetone reduced diclofenac-induced gastric and small intestinal injuries, from 9.5 ± 8.5 in the placebo group to 2.6 ± 3.2 in the geranylgeranylacetone group as evaluated by capsule endoscopy (p = 0.027) [57]. Their data were obtained in a double-blind, randomized, cross-over study where subjects were treated with diclofenac and omeprazole in the presence or absence of geranylgeranylacetone (300 mg/day) for seven days; however, when their analysis was confined to small intestinal injuries, the difference between the two groups was not significant. Geranylgeranylacetone is a gastric mucosal protective agent that is widely used in Japan and other Asian countries for the treatment of gastritis and gastric ulcers [58,59]. Moreover, Shiotani *et al.* also evaluated geranylgeranylacetone for ASA-induced small intestinal injury [29]. They screened twenty healthy volunteers by capsule endoscopy. All subjects were randomly divided into a control group (n = 10) and a geranylgeranylacetone group (n = 10). All eligible subjects were administered low-dose enteric-coated ASA (100 mg/day) for a period of seven days, and the geranylgeranylacetone group received geranylgeranylacetone (300 mg/day). This double-blind, randomized, controlled study found no difference in the incidence of small intestinal injuries between the control and geranylgeranylacetone groups. Table 1 shows a summary of these six trials that used capsule endoscopy to evaluate small intestinal injury. 

Recently, Marchbank *et al*. reported that pacific whiting fish hydrolysate prevented indomethacin-induced permeability [60]. The study did not employ capsule endoscopy. Other studies have shown that fish hydrolysate is beneficial for a variety of gastrointestinal conditions, and one study reported that fish hydrolysate can stimulate the proliferation and migration of HT29 cells *in vitro* [61]. Data for this last study were obtained in a double-blind, randomized, cross-over study where subjects were treated with indomethacin (50 mg/day) for five days in the presence or absence of fish hydrolysate starting two days prior to indomethacin. 

## Conclusions

Although these trials found that misoprostol, rebamipide, metoronidazol, sulfasalazine and fish hydrolysate were effective agents against the development of tNSAID-induced small intestinal injury, the inherent limitations of these studies preclude the drawing of any firm conclusions. First, most of these studies were underpowered and included only a small number of healthy subjects. Second, the short-term NSAID treatment tested is not typical to the clinical setting, where long-term NSAID therapies are the norm. In long-term NSAIDs therapies, the adaptation towards NSAIDs might occur or the polymorphism of COX-1 gene might influence on the NSAIDs induced small intestinal injuries. Misoprostol was the only drug verified in one study to effectively reduce ASA-induced small intestinal injuries. However, the number of subjects used in that study was too low to establish the therapy against ASA-induced small intestinal injuries. Therefore, further extensive studies are clearly required to ascertain the beneficial effect of these drugs.

## Disclosures

All authors have no conflict of interest.

**Table 1:** Studies using capsule endoscopy to evaluate concomitant therapy against NSAID-induced small intestinal injuries.

Reports	Patients	Drop-out	Study design	NSAID	Evaluated drug	Period	Evaluated injuries	Ratio of subjects with injuries		Evaluation
								Control	Treatment	
Fujimori *et al*. [23]	34	4	single-blind	diclofenac	misoprostol	14	mucosal break	53% (8/15)	13% (2/15)	effective
Niwa *et al*. [25]	10	0	double-blind, cross over	diclofenac	rebamipide	7	mucosal break bleeding, redness	80% (8/10)	20% (2/10)	effective
Fujimori *et al*. [24]	80	8	double-blind	diclofenac	rebamipide	14	mucosal break, denuded area	63% (24/38)	47% (16/34)	injuries decreased
Niwa *et al*. [57]	10	0	double-blind, cross over	diclofenac	geranylgeranyl-acetone	7	mucosal break bleeding, redness	40% (4/10)	10% (1/10)	no statistical difference^a)^
Shiotani *et al*. [29]	20	0	double-blind	aspirin	geranylgeranyl-acetone	7	mucosal break	80% (8/10)	100% (10/10)	no difference
Watanabe *et al*. [18]	11	4	case series	aspirin	misoprostol	56	mucosal break red spots	91% (10/11)	43% (3/7)	effective

Note: a) geranylgeranylacetone reduced diclofenac-induced gastric and small-intestinal injuries in all.
